# ACE2 immunohistochemistry in salivary and tracheal glands related to age

**DOI:** 10.1186/s13104-022-06031-1

**Published:** 2022-04-21

**Authors:** Makoto Nogami, Tomoaki Hoshi, Yoko Toukairin, Tomomi Arai, Tadashi Nishio

**Affiliations:** Department of Legal Medicine, Teikyo University School of Medicine, 2-11-1, Kaga, Itabashi-ku, Tokyo, 173-8605 Japan

**Keywords:** ACE2, SARS-CoV-2, COVID-19, Immunohistochemistry, Salivary gland, Tracheal gland

## Abstract

**Objective:**

SARS-CoV-2 is the cause of COVID-19, the rapidly spreading pandemic. When SARS-CoV-2 enters the target cells in the respiratory system, the spike glycoprotein binds to a cellular receptor angiotensin converting enzyme 2 (ACE2). The susceptibility to infection in individuals under 20 years of age is approximately half that of adults aged over 20 years. In this study, we investigated the immunohistochemical protein expressions of ACE2 in mandibular salivary glands and tracheal glands from forensic autopsy specimens covering adults and children.

**Results:**

The ACE2 immunohistochemistry of autopsy specimens was performed, and the percentages of the immuno-positive areas in the cell layers of the glands were calculated. Our results demonstrate that the ACE2 positivity in mandibular salivary gland and tracheal glands showed the statistically significant decrease with the increase of age, which indicates that the susceptibility of aged individuals to SARS-CoV-2 may be due to various factors including but not limited to ACE2 protein expressions.

## Introduction

The novel coronavirus SARS-coronavirus 2 (SARS-CoV-2) is the cause of the coronavirus disease 2019 (COVID-19), the rapidly spreading pandemic. When SARS-CoV-2 enters the target cell, the spike (S) glycoprotein binds to a cellular receptor angiotensin converting enzyme 2 (ACE2) [1]. For a full understanding of the susceptibility for SARS-CoV-2 infection and the role of ACE2 in clinical manifestations of the disease, it is necessary to study the cell type-specific expression of ACE2 in human tissues, both on the mRNA and protein levels [2]. The respiratory system is of special interest because it is the major site of the disease [3]. The expression of ACE2 gene in nasal epithelium is lower in young children compared with adults [4]. In this study, we investigated the immunohistochemical protein expressions of ACE2 in salivary and tracheal glands from forensic autopsy specimens covering adults and children.

## Main text

### Materials and methods

The study was approved by Teikyo University Ethical Review Board for Medical and Health Research Involving Human Subjects (Approve No. 20-084). The profiles of human autopsy cases in this study are as follows: totally, 47 cases (male:female is 2:1) were studied for mandibular and bronchial glands, in which 37 cases were studied for both glands, whereas 5 cases were studied for either one of the glands. The ages range from 0 to 95 years old. The postmortem intervals range from 0.5 to 7.5 days. The causes of death are 3 cases of cardiac diseases, 5 cases of respiratory disease, 5 cases of trauma, 4 cases of asphyxia, 6 cases of drowning, 4 cases of burning, 3 cases of intoxication, 13 cases of other causes, 4 cases of unknown causes. None of the cases had the history of COVID-19 infection, and all cases were negative for COVID-19 PCR at the time of autopy.

Human autopsy samples were fixed in 16% formalin, embedded in paraffin, and sectioned. The immunohistochemistry was performed by the BOND RX automated immunostainer (Leica, Germany) using BOND polymer refine detection system [5]. The antibody used was 500 times diluted anti-ACE2 (rabbit polyclonal, ab15348, Abcam, UK). Diaminobenzidine (DAB) was used for the staining. The immunohistochemical positivity was measured using cellSence software (Olympus, Tokyo, Japan). The range of interest was set on the mandibular salivary glands and tracheal glands, and the brown color of DAB was selected. The percentages of the positive areas in the glands were calculated using the cellSense software (Olympus, Japan).

### Statistical analysis

Linear regression test was used for statistical analyses, and graphs were made by Prism 7 (GraphPad Software, San Diego, USA). The p values less than 0.05 were considered statistically significant.

### Results and discussion

**Fig. 1:**
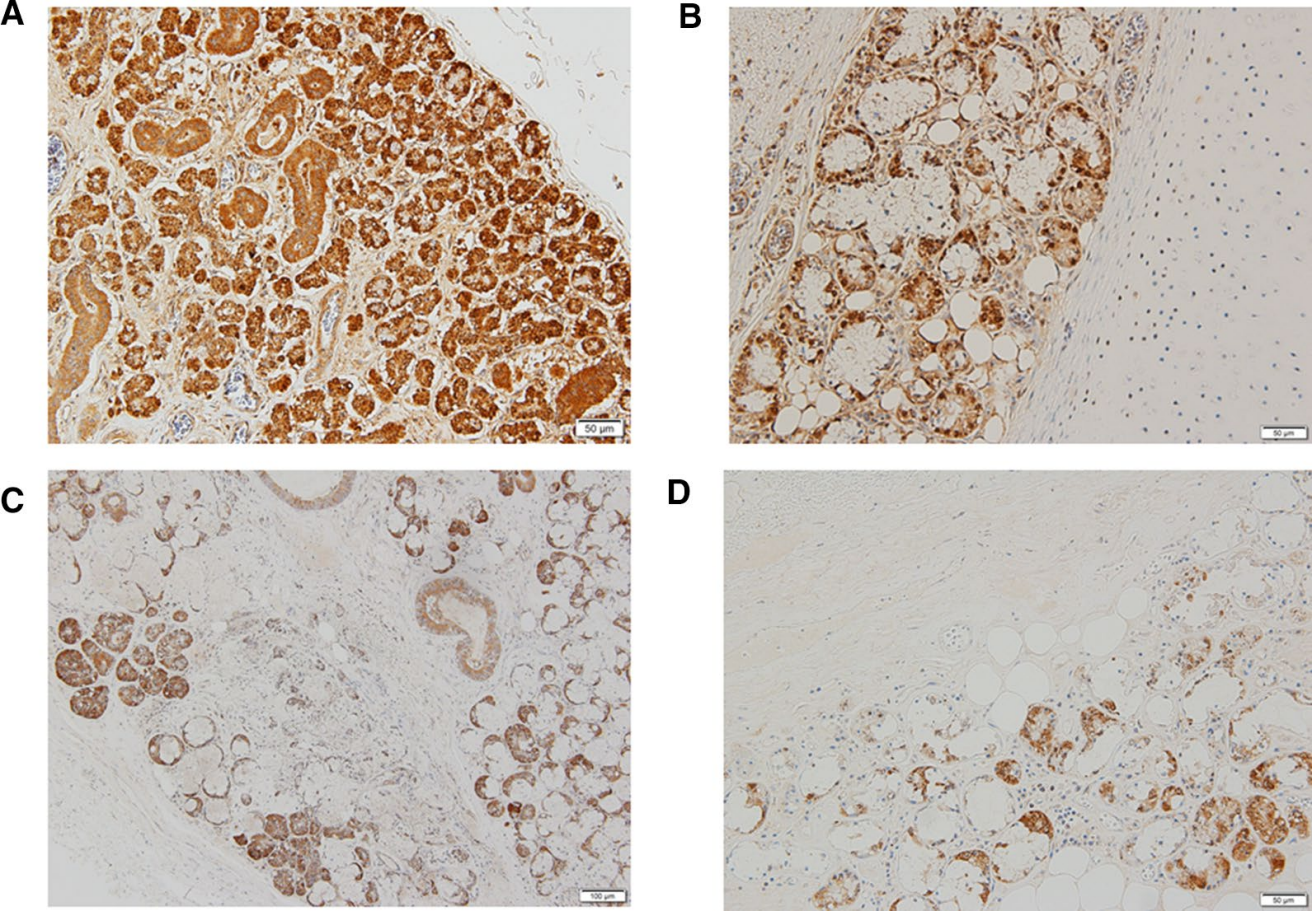
Representative ACE2 immunohistochemistry of mandibular salivary glands (**A**) and tracheal glands (**B**) from 0 year old, whereas mandibular salivary glands (**C**) and tracheal glands (**D**) from 69 years old in contrast. Brown diaminobenzidine-positivity shows ACE2 immunoreactivity. The scales at bottom right show 50 µm

**Fig. 2:**
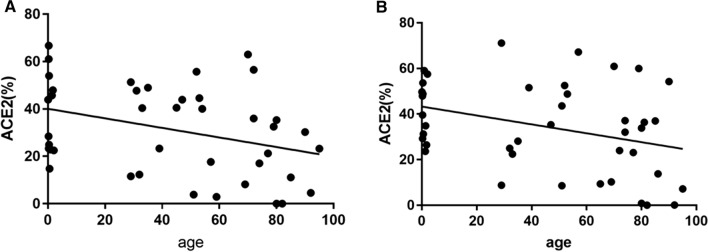
**A** Correlation between ACE2 immunohistochemistry and age for mandibular salivary glands. The statistically significant linear regression line is drawn (n = 42, R^2^ = 0.1265, p < 0.05). Some dots overlap due to close values. **B** Correlation between ACE2 immunohistochemistry and age for tracheal glands. The statistically significant linear regression line is drawn (n = 42, R^2^ = 0.119, p < 0.05). Some dots overlap due to close values

Our study clearly proves the presence of ACE2 immunoreactivity in salivary and tracheal glands (Fig. 1A and B for 0 year old, and Fig. 2A and B for 69 years old). Our results demonstrate that ACE2 is immunohistochemically expressed in salivary glands and tracheal gland cells through all ages studied (Fig. 2A, B). COVID-19 has a characteristic in age distribution. That is, the susceptibility to infection in individuals under 20 years of age is approximately half that of adults aged over 20 years [6]. The ACE2 prevalence in children compared with adults would offer the important information as to the reason why the susceptibility is different in terms of age. Previously, the expression of ACE2 gene in nasal epithelium has been reported lower in young children compared with adults [4]. Since saliva is an important source of SARS-CoV-2 infection [7], we investigated the immunohistochemical expression of ACE2 in submandibular salivary glands as well as tracheal glands as the possible infection sites. Contrary to the above nasal result, our results reveal the novel finding that the ACE2 protein immunoreactivity in mandibular salivary glands (Fig. 2A) and tracheal glands (Fig. 2B) shows the statistically significant decrease with the increase of age. Thus, our results indicate that immunohistochemical ACE2 expressions may inversely correlate with the age. This indicates that the susceptibility of aged individuals to SARS-CoV-2 may be due to various factors including but not limited to ACE2 protein expressions.

### Limitations

Since this is an immunohistochemical study, the functional aspects of ACE2 in the glands are not covered.

## Data Availability

The data sets used and/or analyzed during the current study are available from the corresponding author on reasonable request.

## Abbreviations

ACE2: Angiotensin converting enzyme 2
SARS-CoV-2: SARS coronavirus-2
COVID-19: Coronavirus infection 2019
